# Supplementary material for: Identification of photoperiod-induced specific miRNAs in the adrenal glands of Sunite sheep (Ovis aries)
Source: Front Vet Sci. 2022 Jul 22;9:888207. doi: 10.3389/fvets.2022.888207 (PMC9354845; doi:10.3389/fvets.2022.888207)
Supplement: Supplementary file 1 [file Data_Sheet_1.ZIP › Supplementary file/miRDeep2 file.zip!/miRDeep2 file/Novel_Structure/SPLP42A1/Novel_299.pdf]

Provisional ID	:	NC_019465.2_13886
Score total	:	1.2
Score for star read(s)	:	-1.3
Score for read counts	:	0
Score for mfe	:	0.9
Score for randfold	:	1.6
Score for cons. seed	:	
Total read count	:	27
Mature read count	:	27
Loop read count	:	0
Star read count	:	0

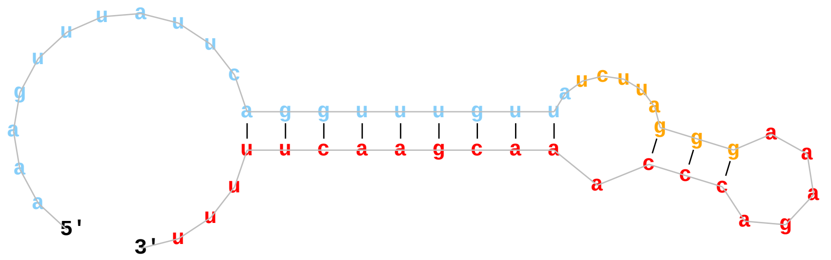

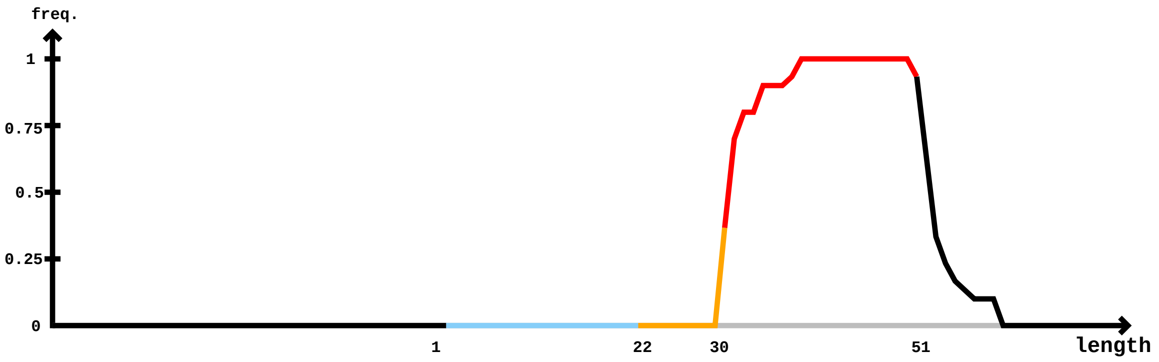

Star Mature

```
5'- caacucuucccacacuauuauaucuauugaguuggccaaaaaguuuauucagguuuguuaucuuagggaaagacccaaacgaacuuuuuggccaacccaauauuuuauug   -3'    exp
    ........................(((((.((((((((((((((((.((..(((((..............)))))..)).)))))))))))))))).)))))........   reads   mm   sample
    ....................................................................aaagacccaaaUgaacuuuuu.....................   11      1    seq
    .....................................................................aagacccaaaUgaacuuuu......................   2       1    seq
    .....................................................................aagacccaaacgaacuuuuug....................   4       0    seq
    .....................................................................aagacccaaacgaacuuuuugg...................   3       0    seq
    .....................................................................aagacccaaacgaacuuuuuggc..................   1       0    seq
    ......................................................................agacccaaacgaacuuuuu.....................   1       0    seq
    ......................................................................agacccaaacgaacuuuuug....................   1       0    seq
    ......................................................................agacccaaaUgaacuuuuuggc..................   1       1    seq
    ........................................................................acccaaacgaacuuuuug....................   1       0    seq
    ........................................................................acccaaacgaacuuuuuggcA.................   1       1    seq
    ........................................................................acccaGacgaacuuuuuggcca................   1       1    seq
    ...........................................................................caaacgaacuuuuuggccaacU.............   1       1    seq
    ............................................................................aaacgaacAuuuuggccaacc.............   1       1    seq
    ............................................................................aaacgaacuuuuuggcUaacc.............   1       1    seq
```
